# Cardanol derived P, Si and N based precursors to develop flame retardant phenolic foam

**DOI:** 10.1038/s41598-020-68910-6

**Published:** 2020-07-21

**Authors:** Caiying Bo, Zhongyu Shi, Lihong Hu, Zheng Pan, Yun Hu, Xiaohui Yang, Puyou Jia, Xiaoli Ren, Meng Zhang, Yonghong Zhou

**Affiliations:** 1 Institute of Chemical Industry of Forest Products, CAF, Nanjing, 210042 Jiangsu Province China; 2 National Engineering Laboratory for Biomass Chemical Utilization, Nanjing, 210042 Jiangsu Province China; 3 Key and Open Laboratory on Forest Chemical Engineering, SFA, Nanjing, 210042 Jiangsu Province China; 4 Key Laboratory Biomass Energy and Material, Nanjing, 210042 Jiangsu Province China

**Keywords:** Chemistry, Materials science

## Abstract

A novel eco-friendly halogen-free cardanol-based flame retardant with P, Si, and N on the chain backbone (PSNCFR) was synthesized and incorporated into phenolic foams (PFs). PSNCFR was comprehensively investigated via Fourier transform infrared spectroscopy and nuclear magnetic resonance. PSNCFR endowed PFs with flame retardancy, contributed to generating a composite char defense against flames, and efficiently prevented smoking from PFs. PSNCFR introduction improved the flexural strength of the PFs to approximately 155% of that of pristine PF. PSNCFR-modified PFs displayed a high limiting oxygen index value of 41.9%. The results of cone calorimeter show that the mean heat release rate, mean effective heat of combustion, and total heat release of the PSNCFR-modified PFs reduced by 26.92%, 35.71%, and 31.25%, respectively. In particular, the total smoke production of the PSNCFR-modified PFs decreased by 64.55%, indicating excellent smoke inhibition. As for the mechanism, the condensation and gas phases during pyrolysis were responsible for the synergistic flame retardancy in the modified PFs. The findings demonstrate that PSNCFR can be used in PF preparation to overcome their drawbacks of internal brittleness and flammability.

## Introduction

Biorenewable resources have attracted significant attention owing to growing concern over environmental problems and energy crisis. Cardanol, an agricultural by-product abundantly extracted from cashew nut shell liquid, is a potential non-edible and safe biorenewable resource^1,2^. Cardanol contains multiple functional groups, such as an aromatic ring, a hydroxyl group, and double bonds in the alkyl chain. Therfore, cardanol is considered a promising resource for preparing of a wide range of chemicals^3,4^. Cardanol and its derivatives can be used as stabilizers, antioxidants, chemical intermediates, anticorrosive paints, plasticizers, and resins^5–7^.

Phenolic foams (PFs), characterized by thermal and chemical stability, adhesivity, high thermal insulation, and flame retardancy, have been widely used in electric instruments, petrochemistry, and architecture^8–10^. However, the application of PFs to other areas are limited owing to their relatively low mechanical performance and high flammability. A previous recent study examined cardanol-based^11^, in particular, the effect of cardanol on the mechanical characteristics of phenol–formaldehyde formulations and the role of cardanol’s flexible alkyl side chains as plasticizers to enhance the mechanical properties of PFs. Unfortunately, the presence of flexible chains of cardanol compromises the flame retardancy of PFs, thereby limiting their use in fields requiring fire resistance.

The most important and feasible requirement to promote the further utility of cardanol-based PFs at the industrial scale is to improve their flame retardancy. Since the European Union banned the use of halogenated fire retardants, flame retardants incorporating P^10,12,13^, Si^14,15^ and N^16,17^ have emerged as efficient non-halogenated candidates. To eliminate PF’s current drawbacks, i.e., the intrinsic brittleness and high flammability, this study examines cardanol as a renewable feedstock to replace phenol in PF synthesis^18^. To impart functional properties in cardanol-based PFs, we have incorporated synergistic flame retardant elements into cardanol structures, which can improve the corresponding functional properties of PFs.

In this study, a novel cardanol-based flame retardant containing P, Si, and N (PSNCFR) on the chain backbone was synthesized and incorporated into PFs. Owing to its unique chemical structure, PSNCFR imparted superior mechanical properties in PF, contributed toward forming a hybrid char antiflame defense, and efficiently avoided smoke generation from PFs. Further the mechanical property, morphology, thermal decomposition, fire avoidance, and smoke restriction of PFs with varying PSNCFR concentrations are further discussed. Finally, the corresponding toughening and flame-retardant mechanisms are presented.

## Results and discussion

### Characterization of PSNCFR

**Figure 1:**
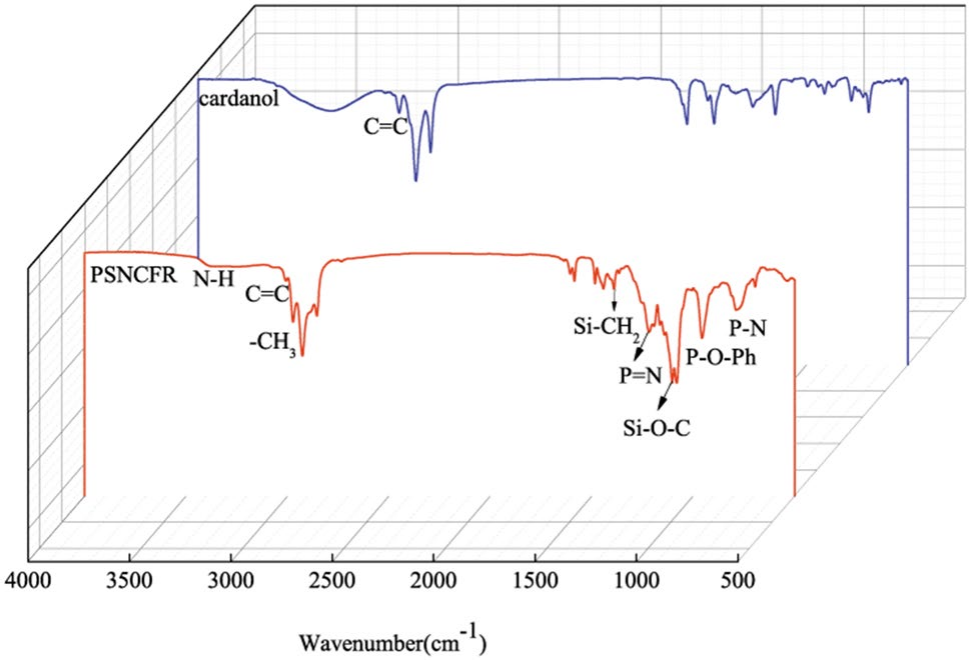
The FT-IR spectra of cardanol and PSNCFR.

Figure 1 shows the Fourier transform infrared (FT-IR) spectra of cardanol and PSNCFR. Clearly, the spectrum of PSNCFR shows absorption at 3,010, 2,926, 2,856, and 1,586 cm^−1^, indicating that PSNCFR contains various functional groups of cardanol^19^. Compared with cardanol, the peaks at 3,346 cm^−1^ (–OH) in the spectrum of PNCFR (see Supplementary Fig. S1 online) almost disappeared, implying that the phenolic hydroxyl groups were converted. The FT-IR spectrum of PSNCFR shows typical peaks at 2,973 cm^−1^ (–CH_3_ of 3-aminopropyltriethoxysilane (APTES)), 1390 cm^−1^ (Si–CH_2_), 1103 cm^−1^ (Si–O–C), and 3,379 cm^−1^ (N–H)^20^ indicating the incorporation of the APTES moieties in PSNCFR. In addition, the peaks at 1,217 and 788 cm^−1^ were assigned to the P=N and P–N stretching vibration^21,22^, and the peak at 957 cm^−1^ was assigned to P–O–Ph. Howerver, the peaks at 600 and 519 cm^−1^ corresponding to the stretching vibration of P–Cl in phosphonitrilic chloride trimer (HCCP)^23,24^ (see Supplementary Fig. S1 online), disappeared, as represented in the supplementary document. The above-mentioned absorptions confirm the reaction of cardanol with HCCP and APTES.

**Figure 2:**
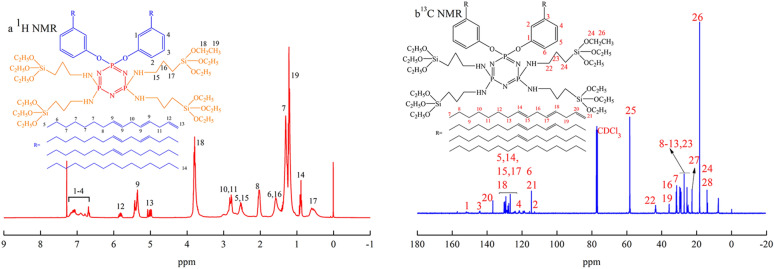
The (**a**) ^1^H and (**b**) ^13^C NMR spectrum of PSNCFR.

The proton nuclear magnetic resonance (^1^H NMR) spectrum of PSNCFR (Fig. 2a), i.e., the peaks at 7.24–6.66 ppm, are attributed to the protons on the benzene ring, and those at 5.83, 5.43–5.34 and 5.07–4.97 ppm correspond to the proton of –CH=CH– in the cardanol moiety. The protons in the—(CH_2_SiOCH_2_CH_3_)—moiety occur at 3.79 ppm (SiOCH_2_), 1.20 ppm (CH_2_CH_3_), and 0.56 ppm (C*H*_2_SiOCH_2_CH_3_). The carbon-13 nuclear magnetic resonance (^13^C NMR) of PSNCFR (Fig. 2b) shows the unsaturated carbons on the aromatic ring and alkyl side chains of the cardanol moiety in the range of 112–157 ppm. The chemical shifts at 35–22 and 14.22 ppm are attributed to the carbons in the methylene and methyl groups of the alkyl side chains of cardanol^25^. The new peaks that appeared at 58.29, 18.28 and 43.29 ppm are attributed to the carbons in the –OCH_2_CH_3_ and N–CH_2_– groups. Phosphorous-31 nuclear magnetic resonance (^31^P NMR) spectrum was also used to elucidate the chemical structures as shown in Supplementary Fig. S2. Because the six monomers of HCCP were mix-substituted by cardanol and APTES, the chemical environment of phosphorus atoms in the same monomer was different for its asymmetric nature^26^. The above mentioned absorptions indicate the formation of flame-retardant PSNCFR.

**Figure 3:**
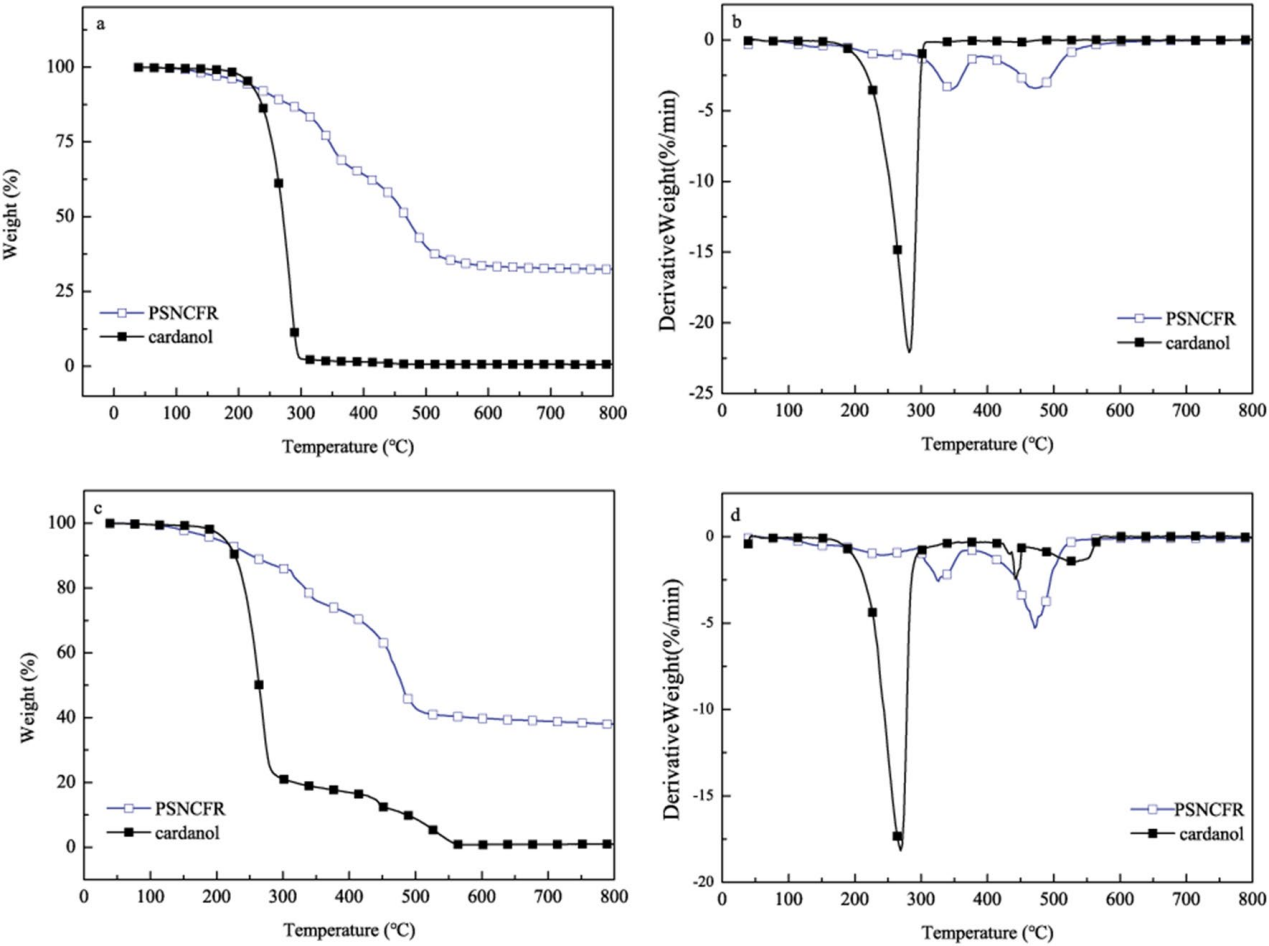
The (**a**) TGA and (**b**) DTG curves of cardanol and PSNCFR under N_2_, (c) TGA and (d) DTG curves of cardanol and PSNCFR under air.

Figure 3 presents the thermogravimetric analysis (TGA) and derivative thermogravimetry (DTG) curves of cardanol and PSNCFR under N_2_ and air. Table 1 shows the initial decomposition temperature (T_i_), temperature at maximum heat weightlessness rate (T_max_), and char yield. Clearly, PSNCFR was thermally decomposed under N_2_ in two stages. In first stage (250–380 °C), the weight loss of approximately 34.34% can be ascribed to the decomposition of oxygen functionalities and aliphatic chains. In the second stage (380–800 °C), the weight loss of 33.15% can be assigned to the pyrolysis of silane, phosphorus moieties, and benzene rings, as well as to the generation of char residues^27^. The thermal degradation of PSNCFR was changed by the introduction of APTES and HCCP moieties. Clearly, the T_i_ and T_max_ of PSNCFR were higher compared with cardanol, and PSNCFR had a higher mass residue than cardanol (32.54% vs 0.63%), indicating that PSNCFR was more thermally stable. This is because the introduction of P, N, and Si into the PSNCFR structure enhanced its thermal stability. Figure 3b shows a similar two-stage thermal decomposition for PSNCFR in air. According to the TGA and DTG results in Fig. 3, PSNCFR has weight loss at 100–200 °C. The minor stages are assigned to the release of residual solvent or small molecular weight impurities from the samples.

**Table 1:** TGA data of cardanol and PSNCFR.

Samples	Degradation step (no.)	Degradation step (°C)		Mass loss (%)	Residual mass (%)
		T_i_	T_max_		
N_2_					
Cardanol	I	253.0	282.6	99.37	0.63
PSNCFR	I	271.6	344.7	34.34	–
	II	386.7	472.1	67.49	32.51
Air					
Cardanol	I	237	269.3	99.06	0.94
PSNCFR	I	298.8	325.5	26.06	–
	II	376.3	471.5	62.03	37.97

### Physical properties of pristine PF and PSNCFR-modified PFs

**Figure 4:**
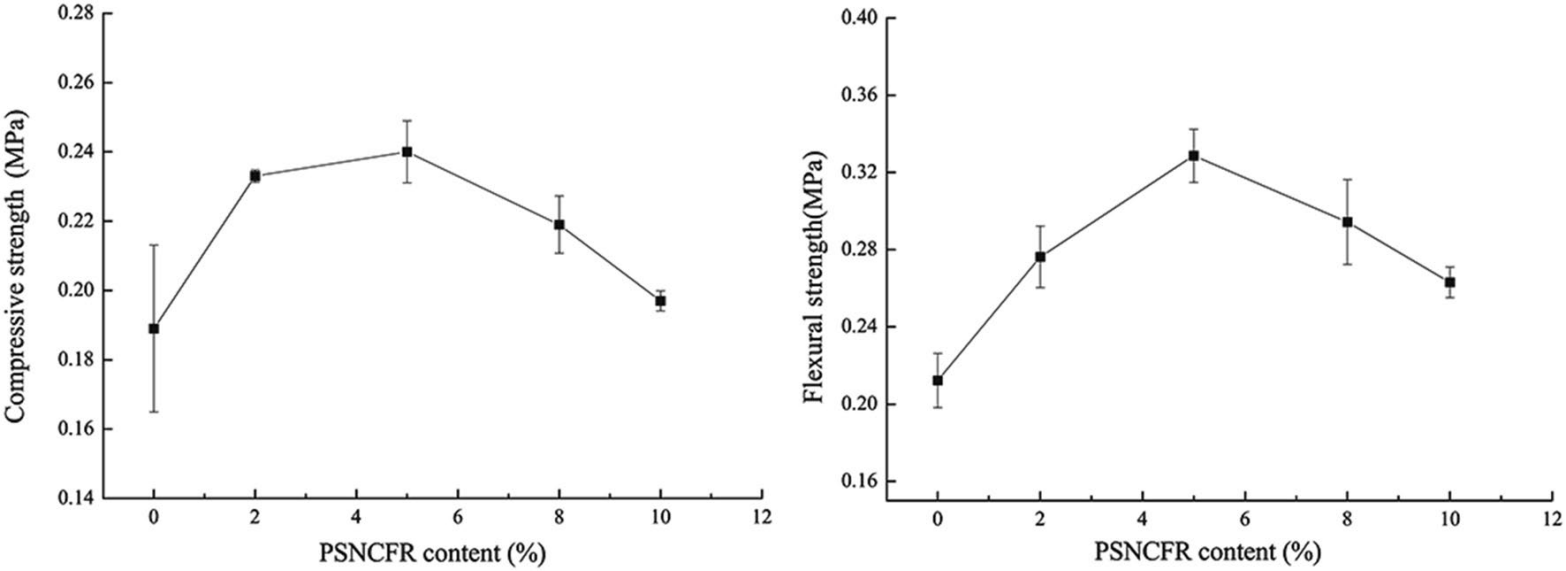
The mechanical properties of pristine and PSNCFR-modified PFs.

**Scheme 1:**
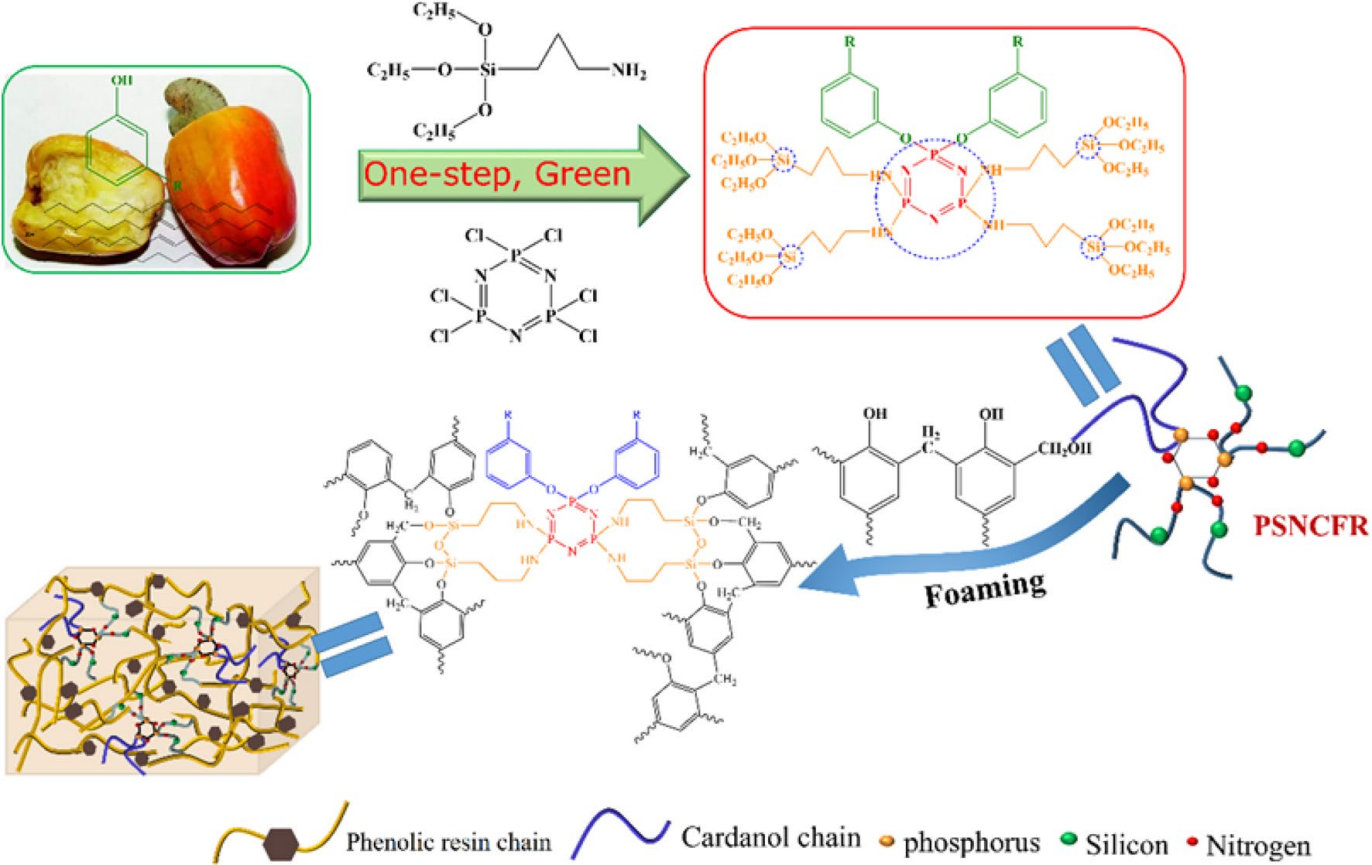
The synthetic route for preparation of PSNCFR and schematic of PSNCFR toughening of PF.

The reinforcing and toughening effects of the PSNCFR-incorporated PFs were investigated via static compressive tensile and flexural tests (Fig. 4). The compressive strength of the modified PFs was first enhanced by increasing the PSNCFR content; it was maximized to 0.240 MPa, which was 26.98% higher than that of pristine PF (0.189 MPa), at 5% PSNCFR in resin. However, further increase in PSNCFR content weakened the compressive strength. When the PSNCFR content was 10%, the compressive strength of the modified PFs dropped but was still higher than that of pristine PF. Moreover, the flexural strength of PSNCFR-modified PFs first increased and then reduced with an increase in PSNCFR content. It maximized at 5% PSNCFR to 0.329 MPa, which was 55.19% higher than that of pristine PF (0.212 MPa). At 10% PSNCFR content, the flexural strength slightly decreased but still outperformed pristine PF due to a condensation reaction between the triethoxysilyl group in PSNCFR and the hydroxyl groups in the phenolic resin linked flexible alkyl side chains to the main chains of phenolic resins^28^. However, with an increase in PSNCFR content, the alkyl side chains enhanced the steric hindrance against the formaldehyde-phenol reaction. Thus, the modified foams were mechanically worsened with excess PSNCFR. The toughening mechanism is illustrated in Scheme 1.

**Figure 5:**
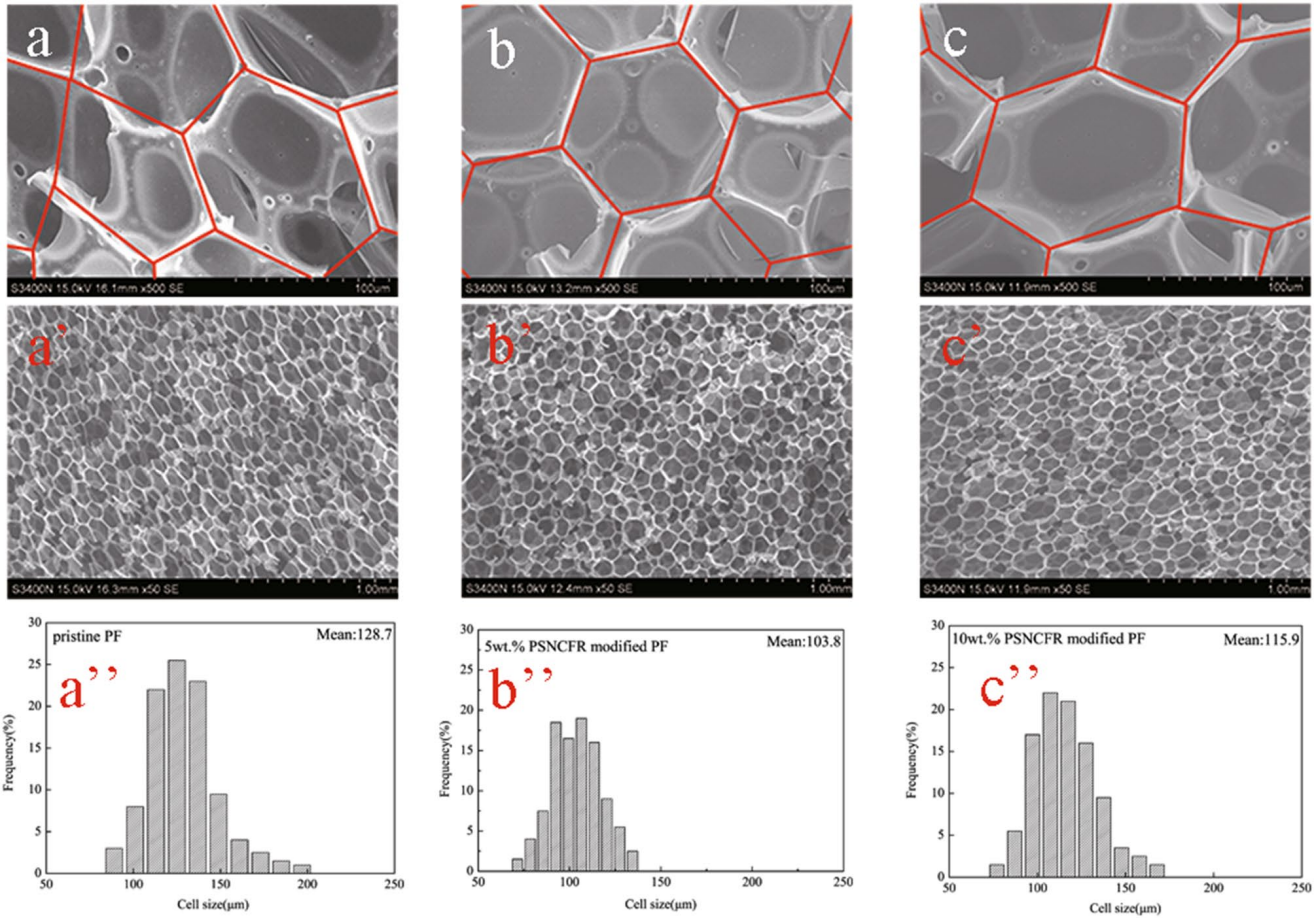
SEM of (**a**,**a**’) pristine, (**b**,**b**’) 5% PSNCFR-modified and (**c**,**c**’) 10% PSNCFR-modified PF, cell sizes distribution of (**a**’’) pristine, (**b**’’) 5% PSNCFR-modified and (**c**’’) 10% PSNCFR-modified PF Cell sizes distribution of pristine and modified foams were computed by Nano Measurer 1.2.0, the software does not require permission to be used and freely available.

The effect of PSNCFR on the microstructure of the PFs was investigated via scanning electron microscopy (SEM, Fig. 5). We computed the sizes of 200 cells of pristine or modified foams from SEM images taken by Nano Measurer 1.2 (Fig. 5). The mean size of pristine PF was 128.7 μm, and the cell size distribution ranged from 83.32 to 202.05 μm. However, for PSNCFR-modified foams, the cell shape was hexagonal and formed a stable honeycomb-like structure, which contrasts with the ellipsoid-like cells exhibited by pristine PF. The cell morphology of PSNCFR-modified foams was more even when the mean cell size was smaller and the cell size distribution was narrower. When the PSNCFR mass fraction was 5% and 10%, the mean cell sizes of the modified foams were 103.8 and 115.9 μm, respectively, with a decline of 19.35% and 9.95% compared with pristine PF, respectively. The presence of irregular cells, a uniform cell distribution, and a stable honeycomb-like structure will result in outstanding mechanical properties^8,29^. These results are similar to those of the mechanical analysis.

### Flame retardancy

**Figure 6:**
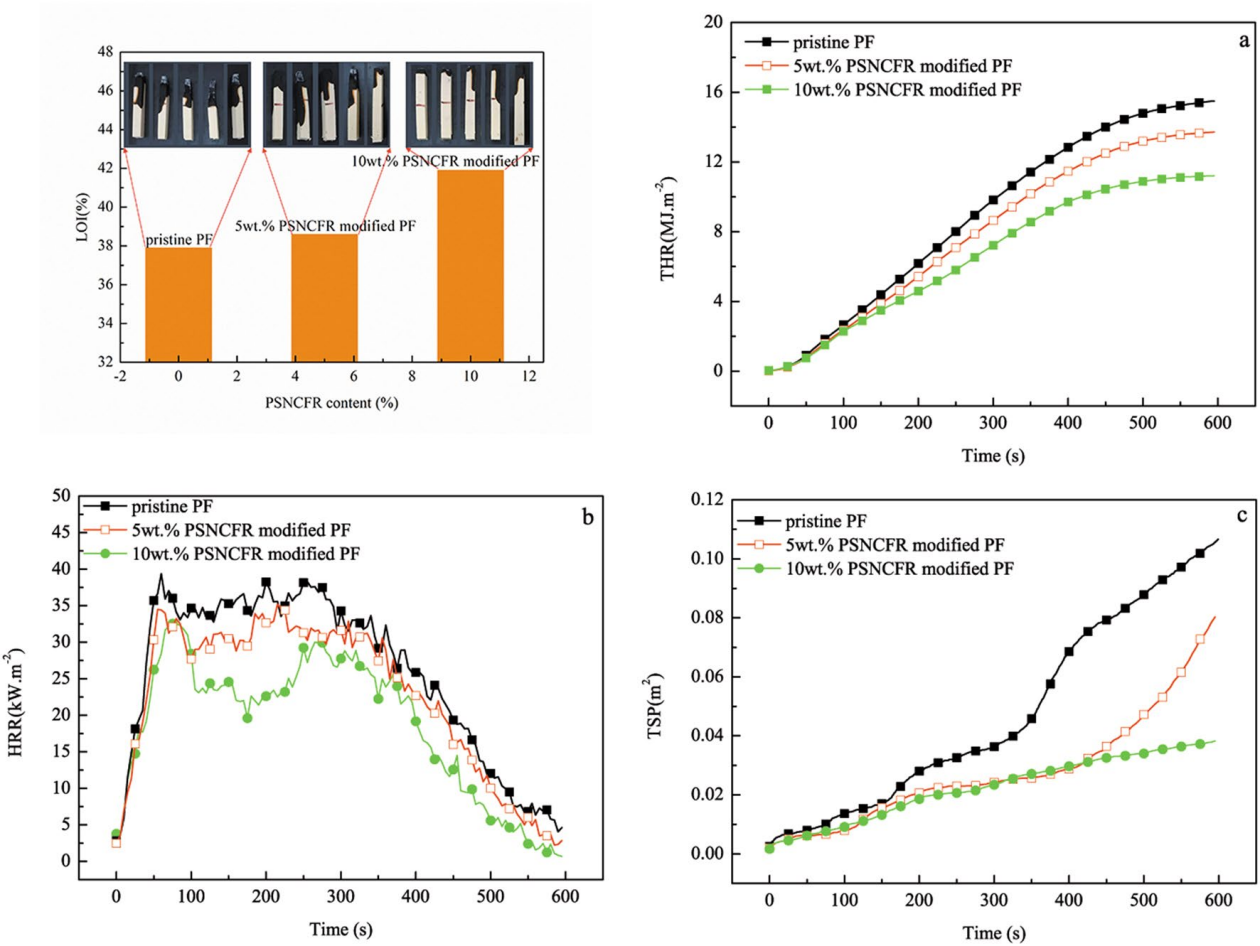
LOI and curves of (**a**) THR, (**b**) HRR and (**c**) TSP of pristine and PSNCFR-modified PFs.

The flame retardancy of the modified PFs was characterized using the limiting oxygen index (LOI) and cone calorimeter test. A higher LOI indicates better flame retardancy. As expected, the use of PSNCFR improved the flame retardancy of the PFs. The LOIs of the PSNCFR-modified PFs added with 5 wt% and 10 wt% PSNCFR were 38.6% and 41.9%, with an increase of ~ 1.85% and 10.55% compared with pristine PF (37.9%), respectively (Fig. 6).

The effects of PSNCFR on the combustion behaviors of PFs were studied by measuring the burning performance parameters using cone calorimetry, including total heat release (THR), heat release rate (HRR), and total smoke production (TSP) (Fig. 6). Moreover, peak HRR (pHRR), mean HRR (mHRR), peak effective heat of combustion (pEHC) and mean EHC are listed in Table 2.

**Table 2:** Cone calorimeter test results.

Samples	HRR (kW m^−2^)		EHC (MJ kg^−1^)		THR (MJ m^−2^)	TSP (m^2^)
	pHRR	mHRR	pEHC	mEHC		
Pristine PF	39	26	7	1.4	16	0.11
5 wt.% PSNCFR modified PF	35	23	6	1.1	14	0.083
10 wt.% PSNCFR modified PF	33	19	6	0.9	11	0.039

HRR, also referred to as fire intensity, measures the heat release rate per unit area from a burning material. pHRR and mHRR are measures of the scale, development, and termination of fire. THR indicates the amount of heat released per unit area from a burning material. Generally, a higher HRR or THR implies a greater risk from fires. Thus, HRR and THR help to more objectively and fully assess burning behaviors^30^.

The THR in our study gradually dropped following an increase in PSNCFR content, and at the load of 10 wt.% PSNCFR, it decreased to 11 MJ/m^2^, which is a drop of 31.25% compared with pristine PF (Fig. 6a).

The HRR of PSNCFR-modified PFs was lower than that of pristine PF (Fig. 6b). The mHRRs of the modified PFs containing 5 wt% and 10 wt% PSNCFR were 23 and 19 kW/m^2^, with a drop of 11.54% and 26.92% compared with pristine PF (26 kW/m^2^), respectively. These data imply that the PSNCFR-modified PFs outperformed pristine PF in terms of flame retardancy.

EHC, defined as the heat released per unit mass of a volatilized material, reflects the combustion efficiency of volatile materials^31,32^. The mEHCs of the modified PFs with 5 wt% and 10 wt% PSNCFR were 1.1 and 0.9 MJ/kg, dropping by 21.43% and 35.71% compared with pristine PFs (1.4 MJ/kg), respectively. At the same external heat flux, the gas phase flame retardancy will reduce EHC more efficiently in comparison with pristine PF.

A lower TSP means a lower smoke risk and longer escape time in fires incidents^33^. The introduction of PSNCFR in PFs considerably reduced their smoke production (Fig. 6c and Table 2). Specifically, the TSP of 10 wt.% PSNCFR-modified PFs decreased by 64.55% when compared with pristine PF (0.039 vs. 0.11 m^2^). These data confirm the excellent smoke inhibition effect of PSNCFR.

### Possible flame-retardant and smoke-suppressant mechanisms

**Figure 7:**
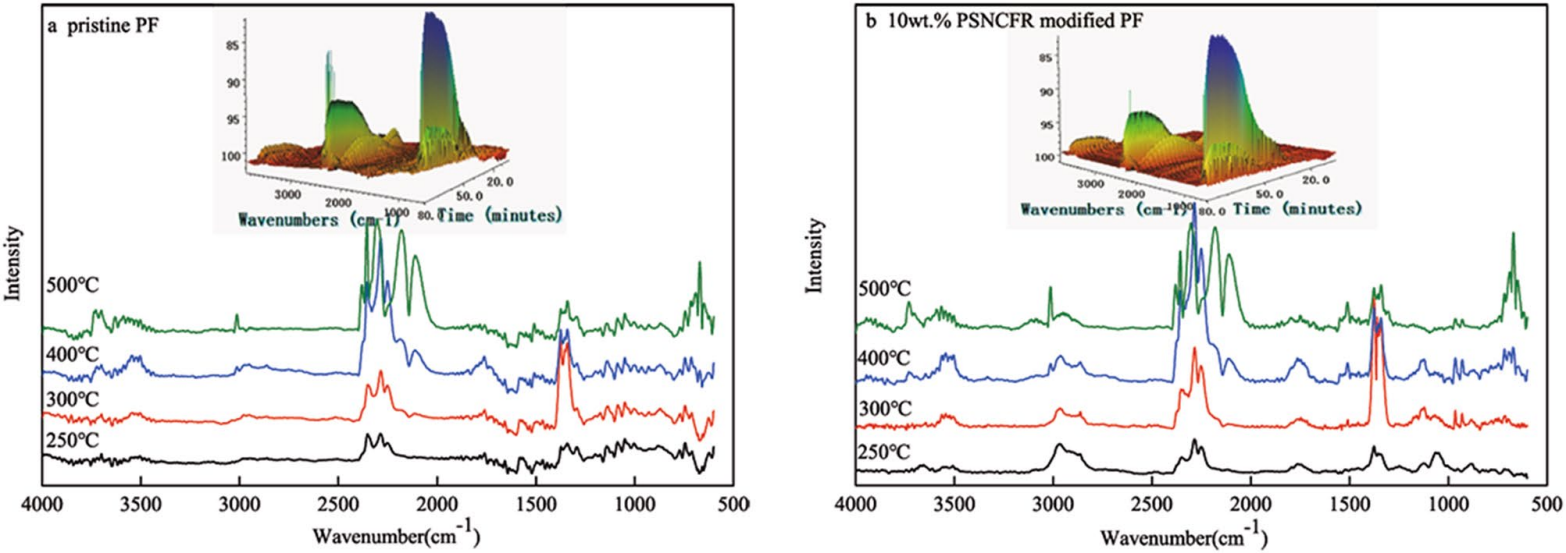
The 3D TGA-FTIR and 2D FT-IR spectra of gas-phase thermal degradation products of (**a**) pristine and (**b**) 10 wt.% PSNCFR modified PF at different thermal degradation stages.

The flame-retardant mechanism of the PFs was probed using TGA-FTIR (Fig. 7). Clearly, the gaseous pyrolysis products of pristine or 10 wt.% PSNCFR-PF mainly showed peaks at 3,500–3,800, 2,800–3,000, 2,200–2,400, 1,000–1,800, and 600–800 cm^−1^. The peak position and intensity varied largely with an increase in temperature, implying that the decomposition products differed with temperature. The species of the products evolved at 250, 300, 400, and 500 °C were confirmed by two-dimensional (2D) FT-IR spectra (base of Fig. 7a). At 250 °C, the main products of pristine PF were minor H_2_O (3,750–3,500 cm^−1^) and CO_2_ (2,350–2,240 cm^−1^)^34^. At 300 °C, the absorbance of H_2_O and CO_2_ was much higher than that at 250 °C, which means that more H_2_O and CO_2_ were emitted. The peaks at 3,100–2,850 cm^−1^ correspond to the C-H stretching vibration. At 400 and 500 °C, the positions of the FT-IR peaks were similar, although the intensity varied slightly.

Different from pristine PF, the FT-IR spectrum of 10 wt.% PSNCFR-modified PFs shows absorption at 925 cm^−1^ (P–O–Ph), 1,120 cm^−1^ (P–O), and 1,245 cm^−1^ (P=N)^35^. It is assumed that the degradation of 10 wt.% PSNCFR formed P-containing compounds, which seized the free radicals in the gas phase and restricted burning^36^. However, the FT-IR spectrum of 10 wt.% PSNCFR-modified PFs did not show Si-containing components, indicating that the decomposition products were at the condensed phase and improved the residual char yield.

**Figure 8:**
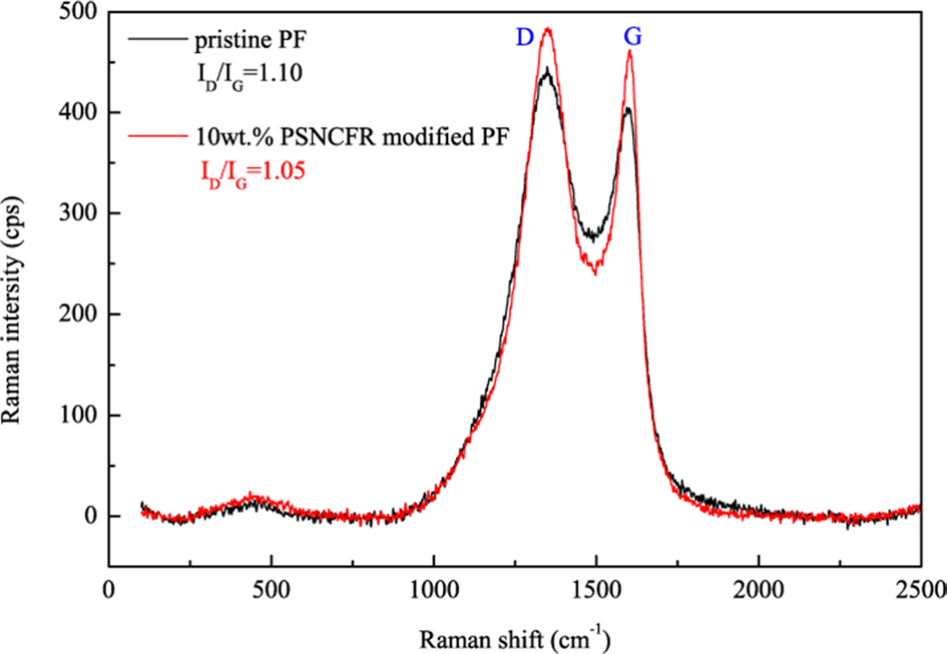
Raman spectra of the char residues after cone calorimeter tests of pristine and 10 wt.% PSNCFR modified PF.

Laser Raman spectroscopy (LRS) characterizes the graphitization degree of carbonaceous substances after combustion^37,38^. The LRS spectra for the outer residual chars of pristine or 10 wt.% PSNCFR-modified PFs after cone calorimetry are shown in Fig. 8. The LRS spectra of carbon signals typically show the D band at ca. 1,360 cm^−1^ and the G band at ca. 1,580 cm^−1^. The intensities of the D and G bands correspond to amorphous carbon and graphitized carbon respectively; accordingly, the D and G band intensity ratio (I_D_/I_G_) characterizes the graphitization extent of residues. A lower I_D_/I_G_ implies a more stable char structure with more intense graphitization and thereby better flame retardancy^39^. The I_D_/I_G_ of 10 wt.% PSNCFR modified PFs was less than that of pristine PF (1.05 vs. 1.10, Fig. 8). This indicates the higher graphitization degree residue of the modified PFs. The introduction of PSNCFR facilitated the char graphitization of PFs after burning, forming a more stable hybrid char to raise the flame retardancy of PFs.

**Figure 9:**
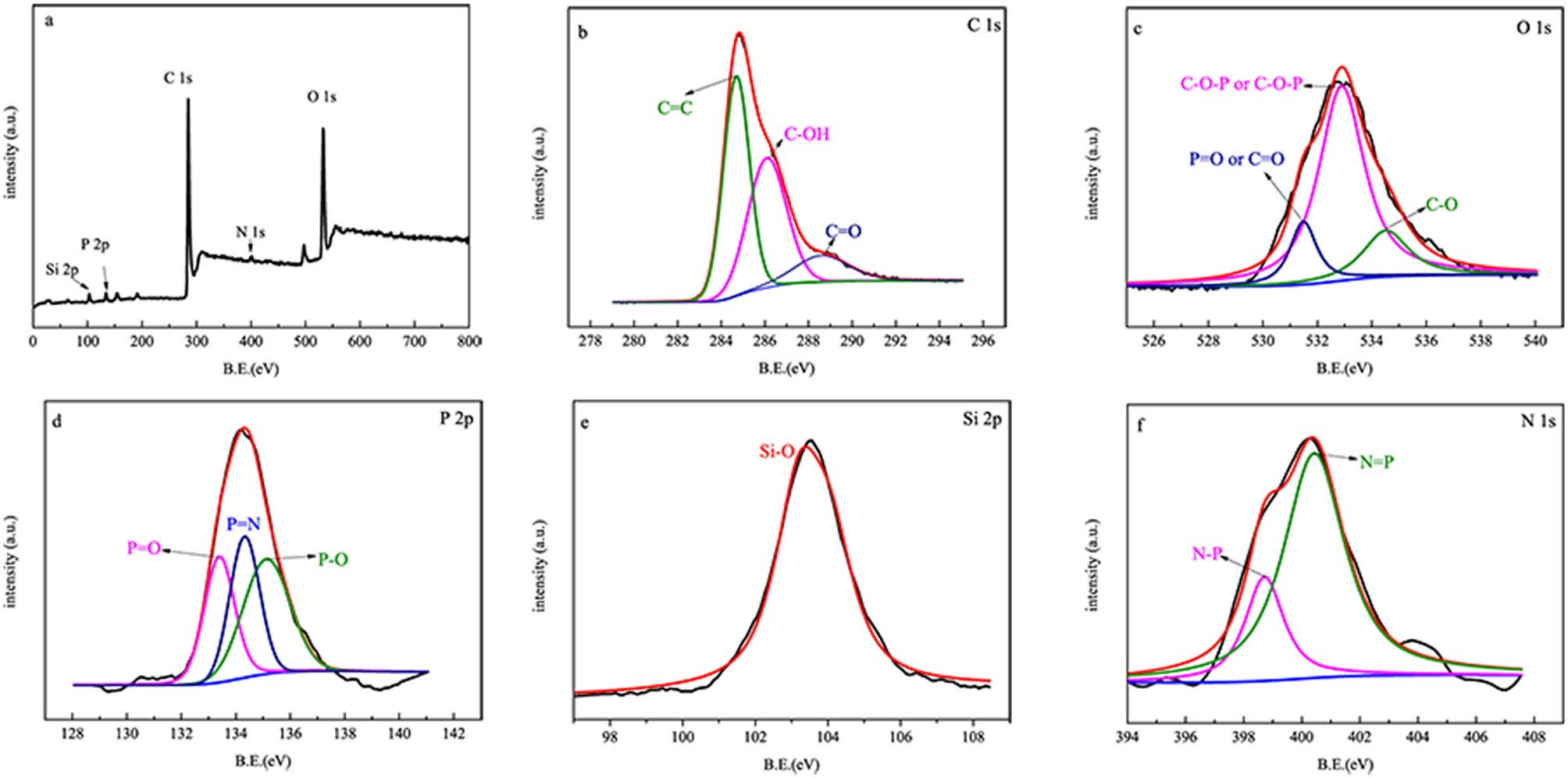
(**a**) XPS wide scanning spectrum, (**b**) C_1s_, (**c**) O_1s_, (**d**) P_2p_, (**e**) Si_2p_ and (**f**) N_1s_ spectra of char residue of 10 wt.% PSNCFR-modified PF.

**Figure 10:**
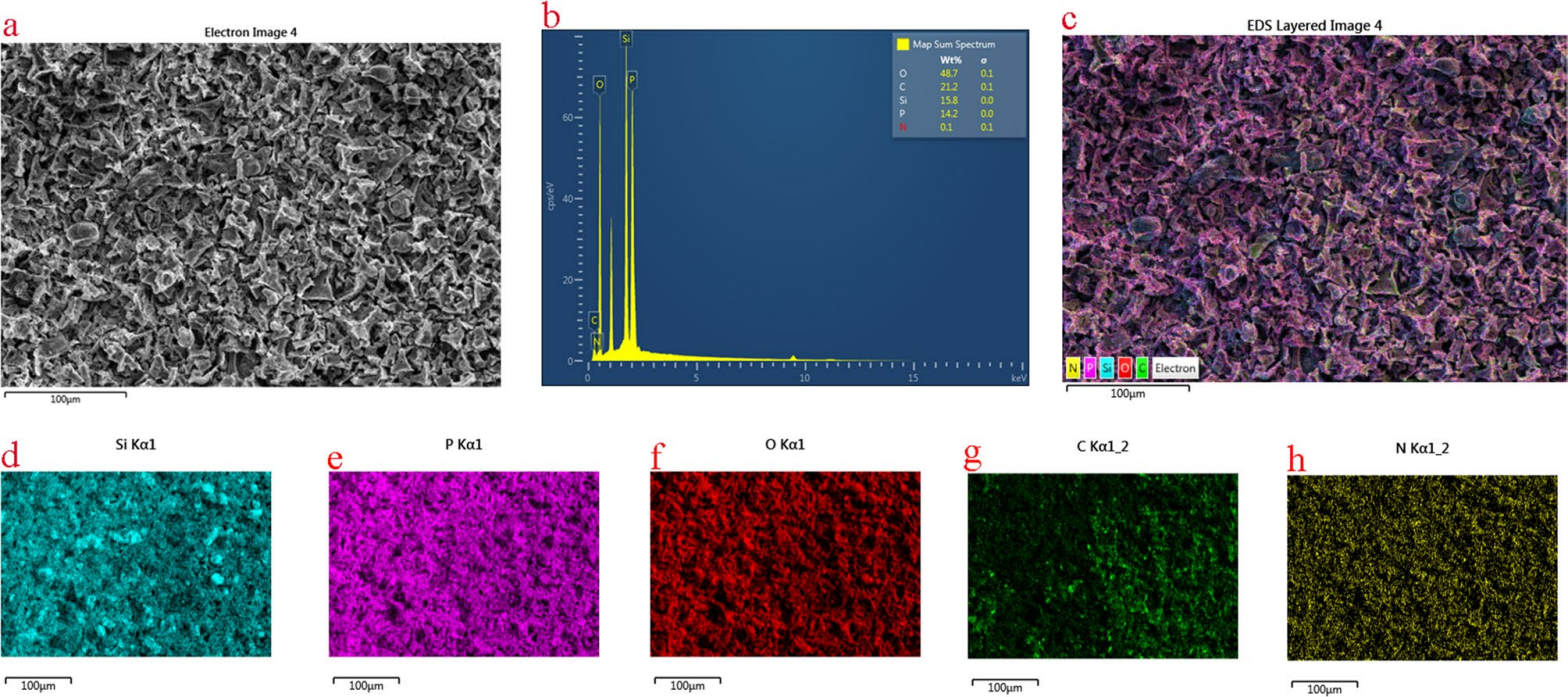
(**a**) Electron image, (**b**) map sum spectrum, (**c**) EDS layered image, (**d**) Si, (**e**) P, (**f**) O, (**g**) C and (**h**) N mapping of char residue of 10 wt.% PSNCFR-modified PF.

To explore the residual char’s structure, the surface elemental composition of 10 wt.% PSNCFR-modified PFs after combustion was characterized using X-ray photoelectron spectroscopy (XPS, Fig. 9) and energy dispersive X-ray spectrometry (EDS) mapping (Fig. 10). Clearly, the residual char of 10 wt.% PSNCFR-modified PFs mainly consists of C, O, N, P, and Si. The C_1s_ XPS spectrum revealed that the following structures could be assigned to C–C and C–H belonging to the aliphatic and aromatic species at 284.6 eV, the peak at 286.0 eV is assigned to C–O, and the bond at 288.6 eV is attributed C = O. The O_1s_ spectrum shows that the bond energy at 531.4 eV is attributed to the P=O or C=O groups, and the peak at a binding energy of 532.8 eV is assigned to C–O–C or C–O–P^40^. The P_2p_ spectrum displays the bond energy of the P=O, P=N, and P–O in the residual char to be 133.4, 134.3, and135.1 eV, respectively^41,42^. The peak in the Si_2p_ spectra at 103.2 eV is attributed to the Si–O–Si structures^43^. In addition, the N_1s_ spectra have two peaks at around 398.7 (N–P) and 400.3 eV (N=P)^44^. The above analysis confirms that the compact char residues isolated inflammable gases and heat and enhanced the flame retardation ability of the PFs.

**Scheme 2:**
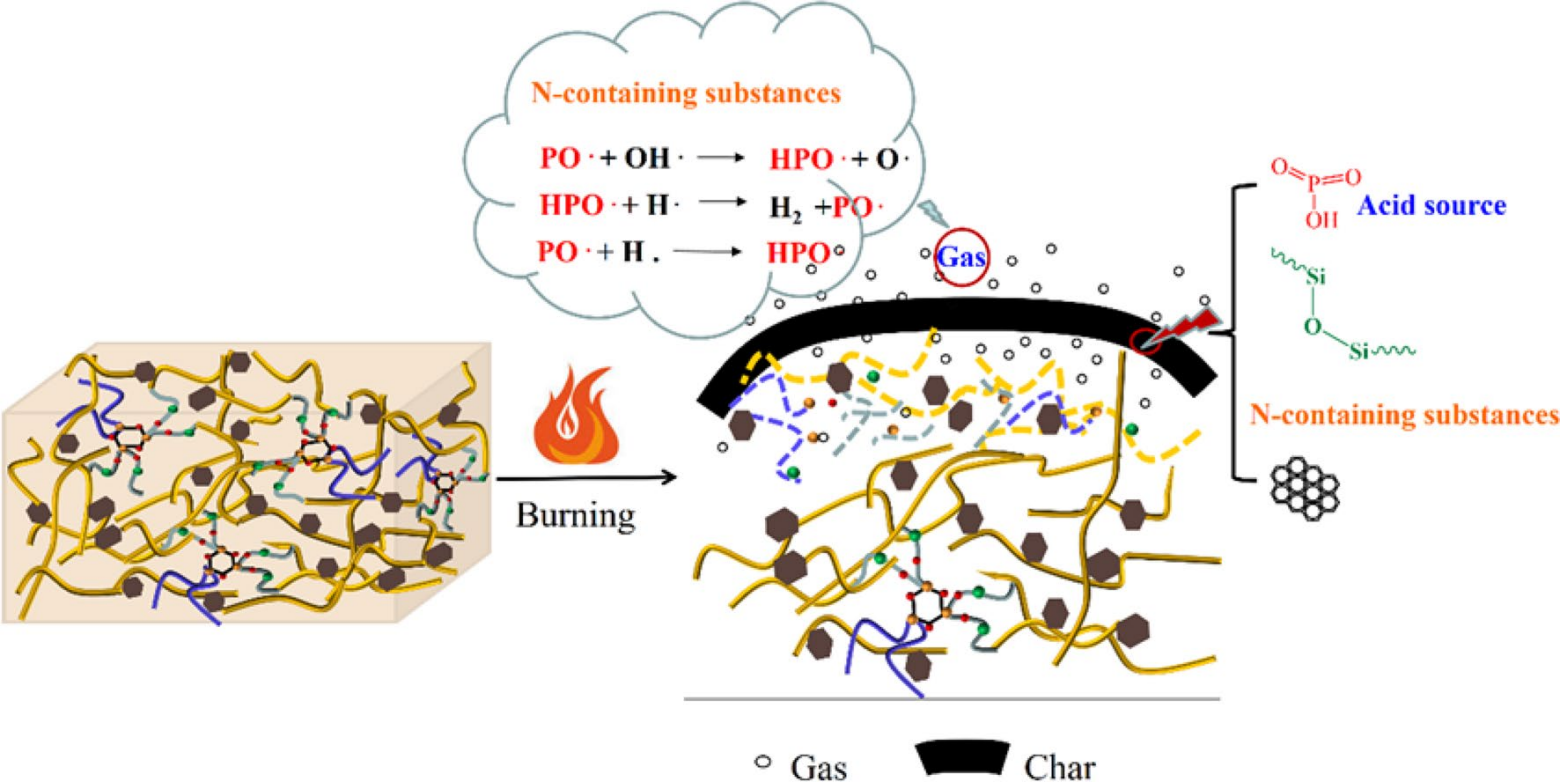
Schematic illustration of the flame retardant mechanism of PSNCFR modified PF.

Based on the above mentioned findings regarding the structure and components of PSNCFR and the thermal stability and flame retardancy of PSNCFR-modified PFs, it is not difficult to understand that the flame-retardant mechanism (Scheme 2). When the PSNCFR-modified PFs were ignited, P- and N-containing flame retardants produced phosphorus-based substances such as phosphoric/polyphosphoric acid and cross-linked phosphorous oxides^40,45^, which, together with the P-,Si-, and N-containing carbon formed during the combustion, contributed to the formation of compact and integral char layers that effectively inhibited thermal decomposition of the modified foam and hindered the heat transfer in the PF matrix^46,47^. Furthermore, the barrier-like char layers delayed the spread of pyrolysis volatiles and eliminated smoke release. Meanwhile, the cyclotriphosphazene groups decomposed to generate PO free radicals with a quenching effect on the gaseous phase. In addition, nitrogenous nonflammable gases originated from the cyclotriphosphazene groups in PSNCFR, which also functioned in the gaseous phase, and the dilution effect further helped in reducing the fire hazard of PFs.

## Conclusions

PSNCFR, a novel cardanol-based eco-friendly halogen-free flame retardant containing P, Si, and N on the chain backbone was synthesized and incorporated into PFs. PSNCFR was fully characterized using FT-IR and NMR. It endowed PFs with flame retardancy, contributed to generating a composite char defense against flames and efficiently prevented smoke generation from PFs. The introduction of PSNCFR improved the flexural strength by approxiamately 155% of that of pristine PF. PSNCFR-modified PFs had a high LOI value of 41.9%. Cone calorimetry tests revealed that the mHRR, mEHC and THR of the PSNCFR-modified PFs were reduced by 26.92%, 35.71% and 31.25%, respectively. In particular, the TSP of the PSNCFR-modified PFs displayed a 64.55% reduction, suggesting high smoke inhibition. As for the mechanism, PSNCFR acted both in the vapor phase and in the condensed phase to restrict exothermic reactions and combustion by changing the degradation route, and thereby enhancing char formation with the least volatile evolution. Hence, PSNCFR can be used in PF preparation to handle the drawbacks of intrinsic brittleness and high flammability.

## Experimental methods

### Materials

Cardanol (~ 95%, Shanghai Meidong Biomaterials Co., Ltd., China), dioxane (≥ 99.5%), triethylamine (TEA, 99%), phenol (> 99%), paraformaldehyde (PFA, ≥ 95%), NaOH (≥ 96%) (Nanjing Chemical Co. Ltd., China); APTES (99%), HCCP (98%) (Aladdin, USA) were all of reagent grade.

### Synthesis of PSNCFR

PSNCFR was prepared as shown in Scheme 1. HCCP (0.033 mol, 11.71 g), TEA (10.12 g, 0.1 mol) and anhydrous dioxane (100 mL) were added into a 500-mL 4-neck flask equipped with a reflux condensation tube, a thermometer and a stirring part. After the flask was heated to 55 °C, a solution of cardanol (21.05 g, 0.067mole) in 100 mL anhydrous dioxane was dropped inside for 1 h. Subsequently, the temperature was raised to 98 °C in an oil bath under N_2_ gas for 24 h (PNCFR). After cooling to 0 °C using an ice bath, TEA (10.12 g, 0.1 mol) was added to the system, followed by dropwise addition of APTES (29.74 g, 0.133 mol) for 30 min. The resulting mixture was heated to 70 °C, and kept for 10 h. The reaction system was processed under reduced pressure to evaporate the solvent, and the residue was filtered and washed with water, leading to the formation of PSNCFR.

### Preparation of pristine and PSNCFR-modified PFs

A mixture of phenol and a NaOH aqueous solution in a 4-neck flask was adjusted to pH 9. After the flask was heated to 70 °C, PFA was added four times (nphenol/nPFA = 1:1.7). Then the temperature was first raised to 85 °C, maintained for 30 min, increased to 90–95 °C, and then maintained for 30 min. Thereafter, PSNCFR at different mass ratios was added and maintained for 30 min of the reaction. Finally, after cooling to 50 °C, the PSNCFR-modified phenolic resin was obtained.

Under rapid stirring, pristine or PSNCFR-modified phenolic resin (100 g), a surfactant (Tween-80/modified silicone oil = 1/1, 5 g) and n-pentane (8.5 g) were blended; then, a curing agent (8.5 g) was added. Thereafter, the mixture was poured quickly into a preheated foaming mold (200 × 200 × 50 mm^3^), followed by 1 h of foaming at 80 °C. Foams (50 ± 1 kg/m^3^) were obtained after cooling and demolding.

### Characterization

The samples were characterized on a Nicolet iS10 FTIR meter (Nicolet Instrument Crop., USA) within 400–4,000 cm^−1^ using a thin KBr pill. ^1^H, ^13^C and ^31^P NMR spectra were obtained using a Bruker AV-300 Advance NMR spectrometer (Bruker Corporation, Germany) at 300 MHz, CDCl_3_ as the solvent, and a ThermoFisher DXR laser Raman spectrometer operated at room temperature with a back-scattering geometry with a 532 nm Ar laser. XPS curves were recorded using a Kratos Axis Ultra DLD (UK) spectrometer to analyse the chemical composition of the residue char after cone calorimetry tests. The STA 409 PC/PG thermal gravimetric analyzer (TGA, Netzsch, Germany) was heated at 10 °C /min to 800 °C under N_2_ and air. On a 409PC/PG thermal analyzer (Netzsch) together with the FTIR device, a sample was heated at 10 °C /min to 800 °C under N_2_ and scanned within 4,000–600 cm^−1^ at a resolution of 4 cm^−1^. Compressive, flexural properties and SEM were analysed as reported before^18^. On the British FTT 0,007 cone calorimeter, a sample of 100 × 100 × 20 mm^3^ wrapped in Al foil was tested at a power of 35 kW/m^2^ as per ISO5660.

## Supplementary information

Supplementary information
