# Supplementary material for: Cardanol derived P, Si and N based precursors to develop flame retardant phenolic foam
Source: Sci Rep. 2020 Jul 21;10:12082. doi: 10.1038/s41598-020-68910-6 (PMC7374163; doi:10.1038/s41598-020-68910-6)
Supplement: Supplementary file 1 — Supplementary information [file 41598_2020_68910_MOESM1_ESM.docx]

Cardanol derived P, Si and N based precursors to develop flame retardant phenolic foam

Caiying Bo, Zhongyu Shi, Lihong Hu, Zheng Pan, Yun Hu, Xiaohui Yang, Puyou Jia, Xiaoli Ren, Meng Zhang, Yonghong Zhou^*^

Institute of Chemical Industry of Forest Products, CAF; National Engineering Lab. for Biomass Chemical Utilization; Key and Open Lab. on Forest Chemical Engineering, SFA; Key Lab. Biomass Energy and Material, Jiangsu Province, Nanjing 210042, China.

^*^Corresponding.author E-mail: zyh@icifp.cn; zhangmeng@icifp.cn; newstar2002@163.com

SI.1 The FT-IR spectra of HCCP and PNCFR





Figure S.1 the FT-IR spectra of HCCP and PNCFR

SI.2 The ^31^P NMR spectra of PSNCFR


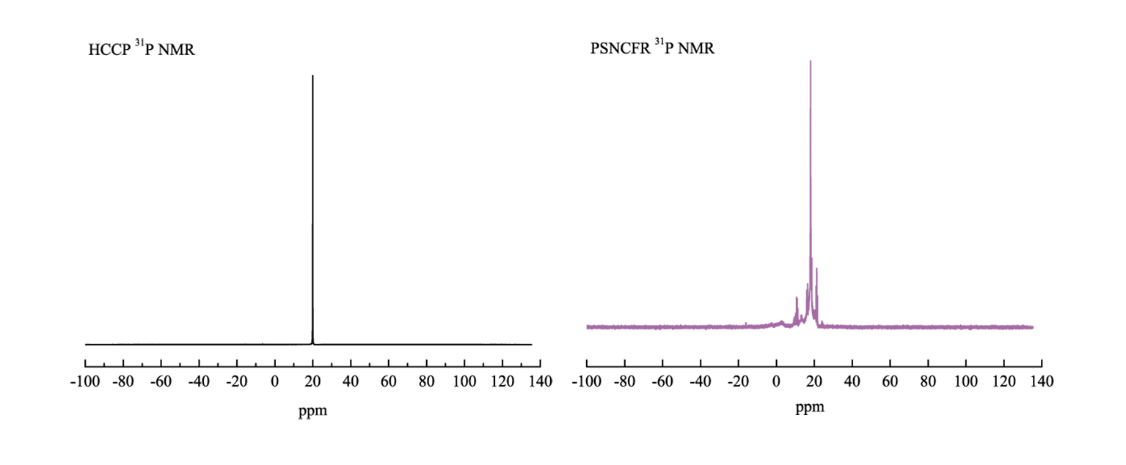


Figure S.2 the ^31^P NMR spectra of HCCP and PSNCFR
